# Inactivation of Serotonergic Neurons in the Rostral Medullary Raphé Attenuates Stress-Induced Tachypnea and Tachycardia in Mice

**DOI:** 10.3389/fphys.2018.00832

**Published:** 2018-07-10

**Authors:** Yoko Ikoma, Ikue Kusumoto-Yoshida, Akihiro Yamanaka, Youichirou Ootsuka, Tomoyuki Kuwaki

**Affiliations:** ^1^Department of Physiology, Graduate School of Medical and Dental Sciences, Kagoshima University, Kagoshima, Japan; ^2^Department of Neuroscience II, Research Institute of Environmental Medicine, Nagoya University, Nagoya, Japan; ^3^Centre for Neuroscience, Discipline of Human Physiology, College of Medicine and Public Health, Flinders University, Adelaide, SA, Australia

**Keywords:** serotonin, medullary raphé, stress, respiration, circulation, body temperature

## Abstract

The medullary raphé nuclei are involved in controlling cardiovascular, respiratory, and thermoregulatory functions, as well as mediating stress-induced tachycardia and hyperthermia. Although the serotonergic system of the medullary raphé has been suggested as the responsible entity, specific evidence has been insufficient. In the present study, we tested this possibility by utilizing an optogenetic approach. We used genetically modified mice [tryptophan hydroxylase 2 (Tph2); archaerhodopsin-T (ArchT) mice] in which ArchT, a green light-driven neuronal silencer, was selectively expressed in serotonergic neurons under the regulation of Tph2 promoters. We first confirmed that an intruder stress selectively activated medullary, but not dorsal or median raphé serotonergic neurons. This activation was suppressed by photo-illumination via a pre-implanted optical fiber, as evidenced by the decrease of a cellular activation marker protein in the neurons. Next, we measured electro cardiogram (ECG), respiration, body temperature (BT), and locomotor activity in freely moving mice during intruder and cage-drop stress tests, with and without photo-illumination. In the intruder test, photo inactivation of the medullary serotonergic neurons significantly attenuated tachycardia (362 ± 58 vs. 564 ± 65 bpm.min, *n* = 19, *p* = 0.002) and tachypnea (94 ± 82 vs. 361 ± 138 cpm.min, *n* = 9, *p* = 0.026), but not hyperthermia (1.0 ± 0.1 vs. 1.0 ± 0.1°C.min, *n* = 19, *p* = 0.926) or hyperlocomotion (17 ± 4 vs. 22 ± 4, arbitrary, *n* = 19, *p* = 0.089). Similar results were obtained from cage-drop stress testing. Finally, photo-illumination did not affect the basal parameters of the resting condition. We conclude that a subpopulation of serotonergic neurons in the medullary raphé specifically mediate stress-induced tachypnea and tachycardia, which have little involvement in the basal determination of respiratory frequency (Res) and heart rate (HR), specifically mediate stress-induced tachycardia and tachypnea.

## Key Points Summary

- The possible role of serotonergic neurons, within the medullary raphé, in physiological responses elicited by stressful events was studied using an optogenetic technique in transgenic mice that expressed ArchT selectively in serotonergic neurons.
- Intruder stress selectively activated medullary, but not dorsal or median serotonergic neurons. This activation was abolished by photo-illumination, as evidenced by the neuron-specific decrease of a cellular activation marker protein, the phosphorylated form of extracellular signal-regulated kinase.
- The inactivation of rostral medullary serotonergic neurons abolished tachypnea induced by intruder and cage-drop stress testing, as well as attenuating stress-induced tachycardia and hyperactivity. However, it did not affect the hyperthermic response or baseline levels of HR, BT, respiration, and locomotor activity.
- These results establish that a subpopulation of medullary raphé serotonergic neurons, which have little involvement in the determination of basal respiratory and HR, specifically mediate stress-induced tachycardia and tachypnea.

## Introduction

Stress causes several physiological responses including tachycardia, tachypnea, hyperactivity, and hyperthermia (Inagaki et al., 2004; Strekalova et al., 2005). These responses are thought to be mediated by medullary raphé neurons, with activation of local neurons leading to subsequent increase in heart rate (HR), blood pressure (Adair et al., 1977; Mccall and Humphrey, 1982), respiration (Lalley, 1986; Besnard et al., 2009), locomotor activity (Dampney, 2015), and body temperature (BT) (Tanaka et al., 2002; Nakamura et al., 2005). Airjet stress-induced tachycardia has been shown to be attenuated by inactivation of the medullary raphé region (Zaretsky et al., 2003b). Psychological stress-induced hyperthermia has also been shown to be attenuated by inactivation of the medullary raphé region (Kataoka et al., 2014). Conversely, the possible role of medullary raphé neurons in the basal regulation of autonomic functions remains controversial. For example, inactivation of medullary raphé neurons does not change basal HR values (Mccall and Harris, 1987) or respiration (Benarroch, 2007); however, it does decrease basal BT (Zaretsky et al., 2003a; Ray et al., 2011).

The raphé nuclei are subdivided into the pons/mesencephalon area (dorsal raphé and median raphé nuclei) and the medulla oblongata (raphé magnus, raphé obscurus, and raphé pallidus) (Holtman et al., 1986). The former has ascending projections to forebrain structures, whilst the latter has descending projections to the brainstem and spinal cord (Holstege and Kuypers, 1987; Aldes et al., 1989; Jacobs et al., 2002). The raphé nuclei contain many serotonin-synthesizing neurons (Dahlstrom and Fuxe, 1964). In the raphé magnus, serotonergic neurons primarily project to the spinal cord and the trigeminal nuclei. In these areas the neurons are involved in the mediation of sensory control (Benarroch, 2014). The raphé obscurus and raphé pallidus project to motor and autonomic nuclei of the spinal cord and the medulla oblongata. These areas mediate respiration, thermoregulation, and cardiovascular function (Azmitia and Gannon, 1986; Holtman et al., 1986; Aldes et al., 1989). These anatomical projections suggest that medullary raphé serotonergic neurons mediate stress-induced physiological responses. In the case of stress-induced cardiovascular and thermoregulatory responses, pharmacological evidence supports the possible contribution of serotonergic neurons. Activation of the serotonin-1A receptor inhibits serotonin release (Gothert, 1990) in the medullary raphé and reduces cardiovascular changes (Nalivaiko et al., 2005) and thermogenesis (Kataoka et al., 2014) during stress. Inhibition of the serotonin-3 receptor in the nucleus tractus solitarius prevents the baroreflex modulation associated with the stress-defense response (Merahi and Laguzzi, 1995). However, there is little information concerning the possible contribution of medullary raphé serotonergic neurons in mediating stress-induced respiratory and locomotor changes. In stressful conditions, cardiovascular, respiratory, and thermoregulatory changes occur simultaneously and cooperate to enable fight-or-flight behavior (Kuwaki, 2011, 2015). Therefore, the simultaneous recording of these parameters is essential for the adequate assessment of the role of serotonergic neurons in stress-induced physiologic responses.

Traditional methods that utilize electrical or pharmacological activation/inactivation of neurons cannot selectively manipulate specific target neurons without affecting the surrounding, non-target, cell types. Increasing utilization of optogenetics has aided in the control of defined clusters of central neurons and thus, has allowed for improved differentiation of their roles in animal behavioral functionality (Yizhar et al., 2011). In this study, we used Tph2-tTA; TetO-ArchT mice, in which archaerhodopsin is selectively expressed in central serotonergic neurons, regulated via a tetracycline-controlled transcriptional activator (tTA) and tTA-dependent promoter (TetO). The archaerhodopsin from the *Halorubrum* strain TP009 is activated via illumination with green light and forms proton pumps that are suitable for complete silencing (Chow et al., 2010; Han et al., 2011; Okazaki and Takagi, 2013).

The aims of the present study were twofold. Firstly, to examine whether serotonergic neurons of the medullary raphé contribute to basal parameters of HR, respiration, BT, and locomotor activity, and secondly, to examine the role of these neurons and their contribution specifically to stress-induced changes in the aforementioned physiological parameters.

## Materials and Methods

### Ethics Approval

All experiments were conducted at Kagoshima University in accordance with the guiding principles for the care and use of animals in the field of physiological sciences published by the Physiological Society of Japan (2015). All efforts were made to minimize the number, and reduce the pain, of animals.

### Animals

Two different types of mice were mated to obtain Tph2-tTA; TetO-ArchT bigenic mice. In the Tph2-tTA transgenic mouse, tTA is expressed under the control of the tryptophan hydroxylase 2 (Tph2) promoter (Ohmura et al., 2014). In the TetO-ArchT knock-in mouse, the beta-actin gene was modified to convey a gene coding tetracycline operator (TetO)- Archaerhodopsin-T (ArchT)-enhanced green fluorescent protein (EGFP) (Tsunematsu et al., 2014). The genotype of Tph2; ArchT mice was identified using polymerase chain reaction of DNA extracted from tail tissue samples. We used a 5′ primer, ACA AGT CCA AGG TGA TCA ACT CCG and a 3′ primer, TCT TCA CGT GCC AGT ACA GG for the Tph2-tTA allele and a 5′ primer, CTG TTC AGC ACC ATC TGC AT and a 3′ primer TCA GCT CGA TGC GGT TCA C for the TetO-ArchT allele. Adult male mice aged >70 days were used. We also used C57BL/6 wild type mice to confirm that non-specific effects of photo-illumination, such as heat, did not affect the physiological responses induced by stress stimuli.

### Confirmation of ArchT (EGFP) Expression in Serotonergic Neurons

To confirm the expression of ArchT in serotonergic neurons, the expression of EGFP in Tph2-tTA; TetO-ArchT mice was examined using immunohistochemistry. The mouse was deeply anesthetized with urethane (2.0 g/kg, i.p.), and perfused with 0.01 M phosphate buffered saline (PBS) followed by 4% paraformaldehyde in 0.01 M PBS (pH 7.4). The brain was then removed and post fixed at 4°C overnight. Coronal sections, including the dorsal raphé (4.5–4.7 mm posterior from bregma), median raphé (4.5–4.7 mm posterior from bregma), rostral medullary raphé (5.5–6.4 mm posterior from bregma), and caudal medullary raphé (6.5–7.5 mm posterior from bregma) were cut at 50-μm thickness using a vibratome and collected in PBS. Floating immunohistochemical staining was performed as follows: sections were sequentially incubated in PBS containing 0.3% Triton-X and 1% normal donkey serum for 30 min, sheep anti Tph antiserum (1/1000, Millipore, AB1541) overnight at 4°C and CF568-conjugated anti sheep IgG (1/200, Biotium) for 90 min at RT in a dark box. Subsequently, sections were incubated with Anti-GFP antibody (1/1000, Nakarai, Japan, #0440484 raised in rat) overnight at 4°C and Cy2-conjugated anti rat IgG (1/400, Jackson Immuno Research) for 90 min at RT in a dark box. Sections were then mounted onto glass slides and examined under a fluorescent microscope.

### Implantation of an Optic Fiber and Devices Used for Physiological Measurements

Mice were anesthetized with isoflurane (2.0%) using a vaporizer for small animals and positioned in a stereotaxic frame (ST-7, NARISHIGE, Tokyo, Japan). A plastic optic fiber (0.2 mm in core-diameter, 0.22 NA; KYOCERA, Kyoto, Japan) was implanted into position 1 mm above of the rostral medullary raphé (on the midline, 6.00 mm posterior from bregma, 4.7 mm deep from brain surface, according to the atlas of Paxinos and Franklin, 2001). A telemetry probe (TA11ETA-F10, Data Sciences International, MN, United States) was also implanted for BT and electro cardiogram (ECG) measurements. To measure respiratory movements, a piezo sensor (D15E60B, Kyoritsu Electric Ind. Ltd, Tokyo, Japan), with nylon-insulated wires (wrapped with silicon), was implanted into the dorsal thorax of a subset of animals. In this last case, output lines from the piezo sensor were connected to connector pins. The connector was attached to the skull with the aid of three stainless screw anchors that were secured using dental cement (Unifast Trad, GC Japan, Tokyo, Japan).

After surgery, mice were given an antibiotic, penicillin G (40,000 U kg**^-^**^1^), and an analgesic (buprenorphine, 0.05 mg kg^-1^). Animals were individually housed and allowed to recover for at least 10 days, under a light–dark cycle (lights on from 7:00 to 19:00) at approximately 23–25°C. Food and water were available *ad libitum*.

After all experimental procedures were completed, the animals were euthanized by an overdose of anesthetic. The position of the optic fiber was confirmed by standard histological techniques. Location of the optic fiber was determined by reference to the Paxinos and Franklin mouse brain atlas (2001).

### Data Acquisition and Signal Processing

Electro cardiogram and BT signals were telemetrically recorded from a chronically implanted telemetry probe, and its receiver (RLA1020, Data Sciences International) that was located under the experimental cage. Locomotor activity was recorded with a passive pyroelectric infrared type motion sensor (AMN 1111, Panasonic Co., Osaka, Japan) that was attached to the ceiling of the experimental cage. The sensor’s output signals (representing the magnitude of each of the animal’s movements) were digitally converted and transferred to a computer. Respiration signals from the piezo sensor were fed into the computer through a cable that was tied in a bundle along with the optic fibers. All signals were digitized (400 Hz for ECG, respiration and timing of photo-illumination; 100 Hz for locomotor activity; and 1 Hz for BT) with an analog-to-digital converter (PowerLab, ADInstruments Inc., Bella Vista, NSW, Australia) and captured with signal processing software (Chart, ADInstruments). The respiratory signal was further filtered (low pass filter, 10 Hz) to calculate respiratory frequency (Res). HR was computed from the ECG signal using Chart software. Other signal analyses were performed using IgorPro (Wavemetrics, Lake Oswego, OR, United States).

Heart rate, Res, BT, and locomotor activity data were averaged for every 30 s to analyze time-related changes.

### Experimental Procedures

All tests were performed during the light period (10:00–19:00). On the morning of the experimental day, a mouse, from its home cage, was transferred to a sound-insulated and temperature-controlled room (23–25°C). An optical cable (Logos, Ibaraki, Japan) was attached to the optical fiber on the animal’s head through a ferrule (KYOCERA) and also connected to a 532-nm DPSS laser (GL532T3-300FC, Shanghai Laser & Optics Century Co., Ltd., China) via a rotary joint (FRJ_FC-FC, Doric Lenses, Quebec, Canada). A flexible electric cable for the piezo sensor was attached to the connector pins on the animal’s head. A minimum period of 4 h was designated for the animal to become habituated to its new environment, and thus, for all physiological parameters to become stable. Continuous recording was then performed until the end of the experiment (from ∼14:00 to ∼19:00). The laser was controlled with an electric stimulator (SEN-3301, Nihon Kohden, Japan) and photo intensity was measured by an optical power meter (PM20, Thorlabs, Newton, NJ, United States).

### Stress Tests

Two types of stress stimuli were applied: one was an intruder test, in which an intruder (male mouse) in a small transparent box was put into the experimental cage for 3 min, preventing physical contact between animals but leaving visual, olfactory, and auditory perceptions unrestricted; the other test was a cage-drop procedure in which a corner of the experimental cage was dropped from a height of 2 cm. Both stimuli were applied when all physiological parameters were at a low and stable resting level and mice were seemingly awake with their eyes open.

**FIGURE 1:**
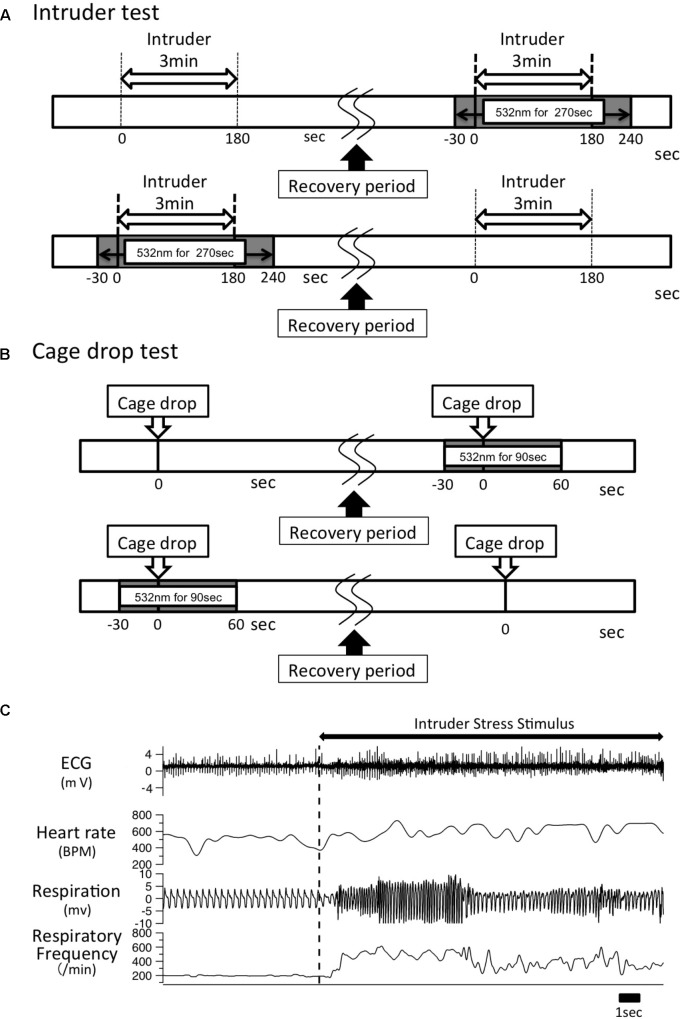
Experimental time line. **(A)** A male intruder mouse was introduced into the resident experiment cage for 3 min. After a recovery period of >60 min and when all the physiological parameters returned to baseline levels, the same intruder stimulus was given along with optical illumination for 270 s, which began 30 s before intruder introduction and lasted until 60 s after the intruder was removed. The order of test sessions (with/without photo-illumination) was randomly assigned to the resident animal. **(B)** A cage-drop stimulus was given at the 0 time point. After the recording parameters returned to baseline level, the same stress was given to the same mouse again with optical illumination for 90 s, which started 30 s before the cage-drop stimulus and ended 60 s after the stimulation. As was the case in the intruder test, the order of test sessions (with/without photo-illumination) was randomly assigned to the animal. **(C)** Typical trace of ECG, respiration signal from a piezo sensor, and calculated heart rate and respiratory frequency. An intruder stimulus was started at the vertical dashed line.

Each stress paradigm was applied twice to each mouse: one with photo-illumination and one without, in a randomized order (**Figures 1A,B**). In our preliminary experiments, we confirmed that two applications of the same stress paradigm (both without photo-illumination) resulted in indistinguishable changes in HR, Res, BT, and locomotion thus indicating little habituation in our experimental protocol (*n* = 3). Photo-illumination (532 nm, 15 mW, continuous) was given for 90 s, which started 30 s prior to the cage drop stimulation, and lasted until 60 s after the stimulus. In the intruder test, the photo-illumination was started 30 s before intruder introduction, kept on for 180 s during intruder-exposure, and lasted for 60 s after the intruder was removed.

### Immunohistochemistry of pERK

Analysis of the expression of pERK was used for two purposes: first to examine whether intruder stress would activate the serotonergic neurons in the dorsal raphé, median raphé, and rostral medullary raphé; and second to confirm that photo-illumination did in fact inactivate the serotonergic neurons. For both purposes, we examined expression of a cellular activation marker protein, the phosphorylated form of extracellular signal-regulated kinase (pERK), in the serotonergic neurons according to a previously published method (Inui et al., 2016). We selected pERK as it has a more rapid and narrow time window (<15 min) compared to other activation markers including c-Fos (≥30 min) – a commonly used activation marker (Antoine et al., 2014).

For the first purpose, we used Tph2-tTA; TetO-ArchT mice, without the optic fiber implantation, that were exposed to the intruder stress for 3 min. The control mouse in this case was exposed to an empty box for 3 min. Immediately after the intruder, or empty box, was removed, the experimental mouse was deeply anesthetized with urethane (2.0 g/kg, i.p.), and perfused with 0.01 M PBS followed by 4% paraformaldehyde in 0.01 M PBS (pH 7.4). Coronal sections, that included the dorsal raphé, median raphé, and the rostral medullary raphé were made and processed as described above (see section “Confirmation of ArchT (EGFP) Expression in Serotonergic Neurons”), with the exception being that a rabbit anti pERK antibody (1/400, Cell Signaling Technology 4370S) was used instead of an anti GFP antibody. The anti pERK antibody was visualized using biotinylated anti-rabbit IgG antibody (1/200, Jackson Immuno Research Laboratories Inc., 711-065-152) and streptavidin conjugated Alexa 568 (1/200, life technologies).

For the second purpose, we compared four groups of the optic fiber-implanted animals: (1) stressed but without photo-illumination, (2) stressed with photo-illumination, (3) no stress and no photo-illumination, and (4) no stress with photo-illumination. The optic fiber was implanted only into the rostral medullary raphé because pERK was found to have increased only in the medullary raphé, not in the dorsal or median raphé (see section “Results”). The intruder male mouse, confined to a small cage, was introduced to the cage of the resident mouse (male Tph2-tTA; TetO-ArchT bigenic mouse) when the resident’s locomotor activity was at a low, resting level but its eyes were open. The intruder was removed 3 min after introduction. Photo-illumination was given 30 s before intruder introduction and kept on for 180 s during intruder-exposure. Immediately after the intruder was removed, the experimental mouse was deeply anesthetized with urethane (2.0 g/kg, i.p.), and processed as described above. The number of pERK and Tph positive cells were counted in one section per animal – in which the track of the optical fiber was most strongly observed.

### Statistical Analysis

Stress-induced changes in HR, respiration rate, and BT over the time course (0–4 min and 4–20 min) were calculated as area under the curve (AUC) over the baseline values. Baseline values were defined as the average of 60 s prior to stress stimulation. The response magnitude of locomotor activity was calculated as the average over the time course (0–4 min and 4–20 min). Repeated measures two-way ANOVA, followed by Sidak’s multiple comparisons test, was used to compare the number of pERK and Tph double positive cells, induced by stress or no stress, in the dorsal, median, and medullary raphé. One-way ANOVA, followed by Bonferroni’s multiple comparisons test, was used to compare the number of pERK-positive cells among the four treatment groups (with/without stress × with/without photo-illumination). A paired *t*-test was used to analyze the differences between values from the photo-illumination trial and from the no photo-illumination trial, as recorded in the same mouse. Data is presented as mean ± SEM. Differences were considered significant at *p* < 0.05.

## Results

### Selective Expression of ArchT-EGFP in the Raphé Serotonin Neurons in Tph2; ArchT Transgenic Mice

**FIGURE 2:**
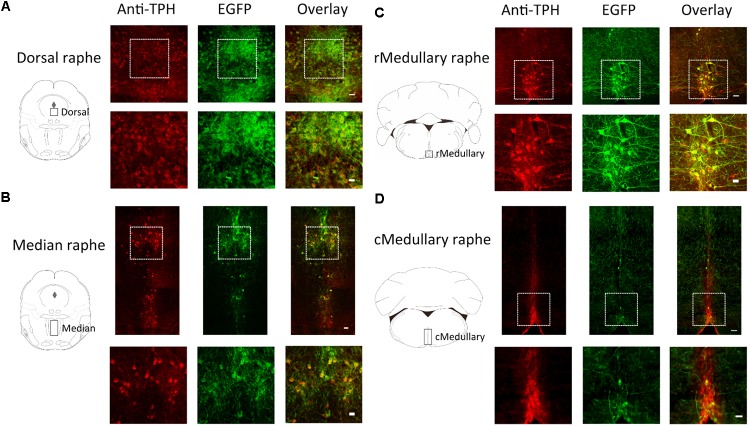
Selective expression of ArchT-EGFP in serotonin neurons in Tph2; ArchT transgenic mice. Immunohistochemical analyses revealed that ArchT-EGFP was specifically expressed in serotonin neurons located in the dorsal raphé **(A)**, median raphé **(B)**, rostral medullary raphé **(C)**, and caudal medullary raphé **(D)** in the Tph2; ArchT transgenic mice brain. Observation areas are indicated by rectangles in the coronal section schemata in the left panels. Tph-immunoreactive neurons (left, red) and ArchT-EGFP-immunoreactive neurons (middle, green) were located in the raphé regions. Overlay image (right) shows specific expression of ArchT-EGFP in serotonin neurons. Bottom row represents higher magnifications of the regions enclosed by the squares in the top row. Bars in the upper and lower rows indicate 50 μm and 25 μm, respectively.

Serotonin neurons in both the pons/mesencephalon area (dorsal raphé and median raphé nuclei) and the medulla oblongata (medullary raphé) of Tph2-tTA; TetO-ArchT mice specifically expressed ArchT, as confirmed by double-labeled immunohistochemistry (**Figure 2**). We used Tph as the marker of serotonin neurons and examined their co-localization with ArchT-EGFP-expressing cells in the three areas. An anti-GFP antibody was used to detect ArchT-EGFP fusion proteins. The merged picture shows that ArchT-EGFP was exclusively observed in serotonin neurons in Tph2-tTA; TetO-ArchT mice, with few displaying ectopic expression.

In a quantitative analysis, we counted cell numbers in the area indicated by rectangles in the coronal section schemata of **Figure 2**. In the medullary raphé, we focused on midline located nuclei (raphé magnus, raphé obscurus, and raphé pallidus) but not laterally located serotonin neurons (parapyramidal areas) since we had intended to use single optic fiber to minimize the brain damage (see also **Figure 4A**). The ArchT expression rate (GFP-immunoreactivity/Tph-immunoreactivity) was 73.4% (4403 GFP-positive cells out of 5996 Tph-positive neurons, from 18 slices of 9 mice) in the pontine dorsal raphé, 79.7% (1133/1421) in the median raphé, 71.3% (530/743) in the rostral medullary raphé, and 31.4% (272/866) in the caudal medullary raphé. Almost all GFP-positive cells were Tph-positive (93.6%, 6338 out of 6769), indicating that ArchT-EGFP was specifically expressed in serotonin neurons.

### Intruder Stress Selectively Activated Rostral Medullary Raphé Serotonin Neurons

**FIGURE 3:**
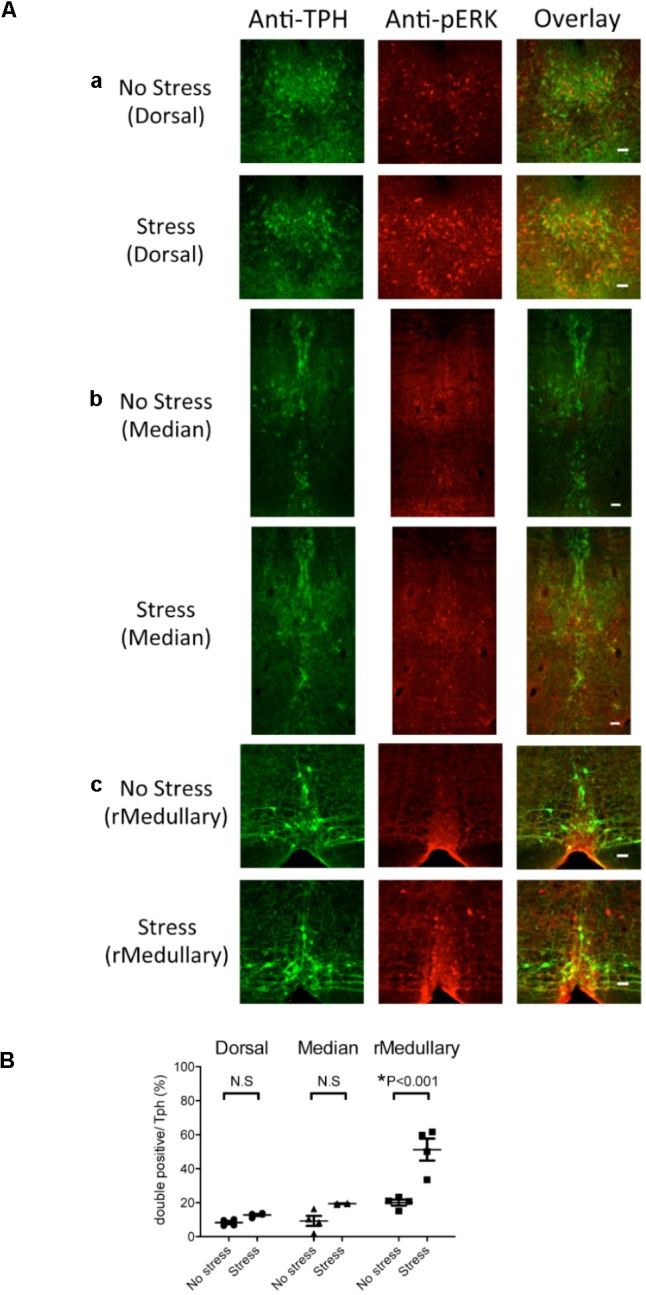
Selective expression of pERK in rostral medullary raphé serotonin neurons induced by intruder stress. Three regions were examined for possible activation by intruder stress using an activation marker, pERK, and an anti-Tph antibody. **(A)** Representative images showing anti-TPH, anti-pERK, and merged staining in the Tph2; ArchT transgenic mice without (top row) or after (bottom row) intruder stress. Examined areas were the dorsal raphé nucleus (a), median raphé nucleus (b), and medullary raphé nuclei (c). See also **Figure 2** for location. Bars in the right rows indicate 50 μm. **(B)** Percentage of the double positive cells (pERK and Tph) in total Tph positive cells. The numbers of animals used were four mice in the no stress group, and four in the stress group.

To examine which raphé nucleus was activated by the intruder stress, we counted an activation marker, pERK, in serotonin (Tph positive) neurons in the dorsal raphé (4.5–4.7 mm posterior from bregma), median raphé (4.5–4.7 mm posterior from bregma), and rostral medullary raphé (5.5–6.4 mm posterior from bregma) (**Figure 3**). Hereafter, we focused on these three raphé nuclei as penetration rate of ArchT-EGFP in the caudal medullary raphé serotonin neurons (∼30%) was smaller than in the other three areas (70–80%, see **Figure 2** and previous section) for unknown reason(s).

The number of Tph positive cells (averaged value from 2 slices/animal) was not significantly different between stressed and no stress groups (dorsal raphé 355 ± 25 vs. 332 ± 27, median raphé 125 ± 9 vs. 120 ± 9, rostral medullary raphé 26 ± 2 vs. 32 ± 2, *n* = 4 for each group). An intruder stress increased the number of pERK and Tph double positive cells only in the rostral medullary raphé (**Figure 3B**) (*p* < 0.001, two-way ANOVA followed by Sidak’s multiple comparisons test), indicating the possible contribution of the rostral medullary raphé in the regulation of stress-induced physiological responses.

### Photo-Illumination Decreased pERK Expression Induced by Stress in Medullary Raphé Serotonin Neurons

**FIGURE 4:**
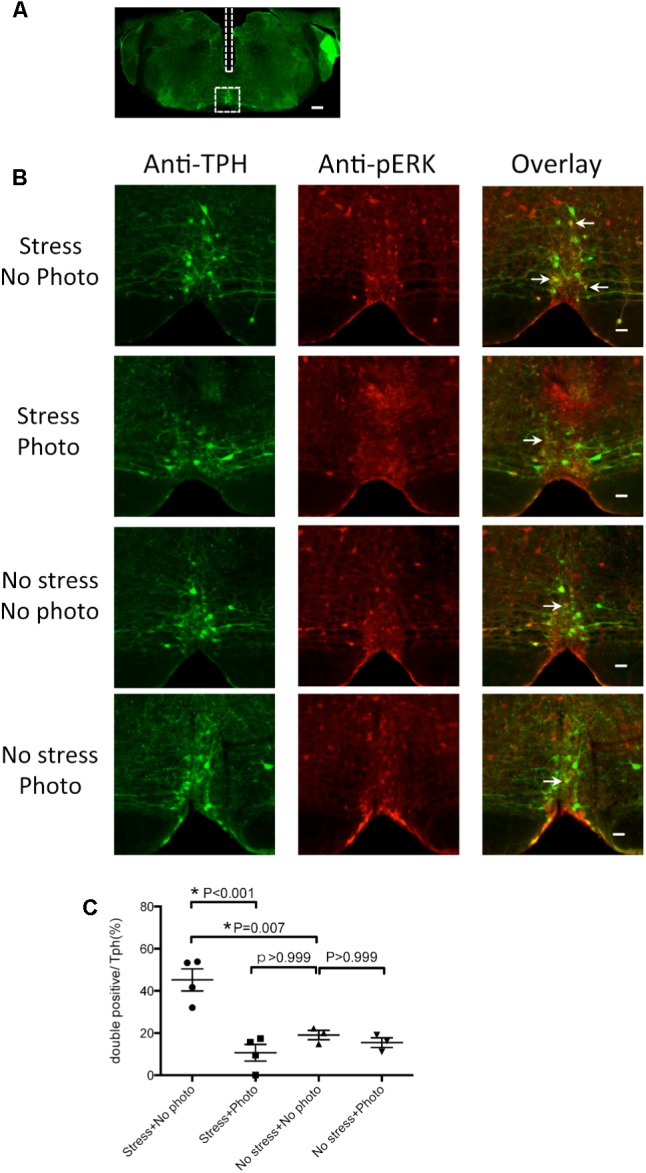
Photo-illumination decreased pERK expression induced by intruder stress in medullary raphé serotonin neurons. **(A)** A low magnification image of the medullary slice showing the optic fiber track (rectangle) and the area for cell counting (square). Bar indicates 300 μm. **(B)** Representative images showing anti-TPH, anti-pERK and merged staining in the medullary raphé of “Tph2; ArchT” transgenic mice after intruder stress without photo-illumination (top row), after intruder stress with photo-illumination (second row), after no stress with no photo-illumination (third row), and after no stress with photo-illumination (bottom row). Arrows indicate example of double (pERK and Tph) positive cells in the medullary raphé. Bars in the top to bottom right rows indicate 50 μm. **(C)** Percentage of the double positive cells. In each animal, data was taken from 1 slice at the level where the center of the fiber optic track was found. The number of animals used were 4, 4, 3, 3 in stress without photo-illumination group, stress with photo-illumination group, no stress with no photo-illumination group, and no stress with photo-illumination group, respectively.

To confirm photo-inactivation of serotonin neurons, we examined expression of pERK as induced by intruder stress. We compared the four groups of animals: (1) stressed but without photo-illumination (Stress + No Illumination, *n* = 4), (2) stressed with photo-illumination (Stress + Illumination, *n* = 4), (3) no stress and no photo-illumination (No Stress + No Illumination, *n* = 3), and (4) no stress with photo-illumination (No Stress + Illumination, *n* = 3). To analyze neuronal activation, we counted the number of cells that were positive for Tph and the cells that were positive for both pERK and Tph (**Figure 4**). We then calculated the ratio for expression (Tph and pERK double-labeled cells/Tph positive cells). Correct location of the tip of the optical fiber was confirmed in all animals (**Figure 4A**).

The numbers of Tph positive cells in the 4 groups were 32 ± 5, 27 ± 3, 34 ± 6, and 28 ± 5, respectively, for Stress + No Photo, Stress + Photo, No Stress + No Photo, and No Stress + Photo groups. There was no statistically significant difference between the groups, indicating reproducible counting of the Tph positive cells using this method.

Without photo-illumination, the 3 min intruder stress increased pERK in Tph neurons (45.2 ± 5.2 % in Stress + No Photo group vs. 19.0 ± 2.2% in No Stress + No Photo group, *p* = 0.007, one-way ANOVA followed by Bonferroni’s multiple comparisons test), indicating that stress activated the rostral medullary raphé serotonin neurons (**Figure 4C**) and fiber implantation did not affect the result (compare with **Figure 3B**). When photo-illumination was applied during stress, double-positive cells were significantly decreased as compared to the Stress + No Photo group (*p* < 0.001), indicating the photo-inactivation of the serotonin neurons during stress. This inactivation seemed to be almost complete as the ratio of double positive cells in Stress + Photo group (10.6 ± 3.9 %) was not significantly different from that in the No Stress + No Photo group (19.0 ± 2.2%, *p* > 0.999). Photo-illumination at baseline (without stress) did not affect the ratio of double positive cells (19.0 ± 2.2% in No Stress + No Photo vs. 15.5 ± 2.3% in No Stress + Photo group, *p* > 0.999).

It may be of note that the number of pERK positive and TPH negative cells (non-serotonergic active neurons) was not different among the groups (10 ± 4, 4 ± 2, 15 ± 1, and 20 ± 8, respectively, for Stress + No Photo, Stress + Photo, No Stress + No Photo, and No Stress + Photo groups), indicating photo-illumination did not affect the activity of non-serotonergic neurons.

### Effect of Photo-Inactivation of Medullary Raphé Serotonin Neurons on Physiological Responses Induced by Stress Stimuli in Tph2; ArchT Mice

To study the possible contribution of medullary raphé serotonin neurons in the regulation of stress induced physiological responses, medullary raphé serotonin neurons were inactivated by photo-illumination *in vivo* using freely moving Tph2-tTA; TetO ArchT mice. The tip of the optical fiber was stereotaxically placed 1 mm above medullary raphé serotonin neurons, and the correct location was confirmed in all animals after the experiment. The physiological parameters that were measured were HR, Res, BT, and locomotor activity (Act). HR and Res were calculated from tracings of ECG and piezo sensor signal, respectively (**Figure 1C**). Thirty animals were used in this experiment; however, 11 were excluded from data analysis due to incorrect fiber position (*n* = 5) and instrumental failure (*n* = 6). The position of the center of the optic fibers in the physiological experiment described below (*n* = 19) was 5.9–6.4 mm posterior from bregma, corresponding to rostral medullary raphé.

### Intruder Test

**FIGURE 5:**
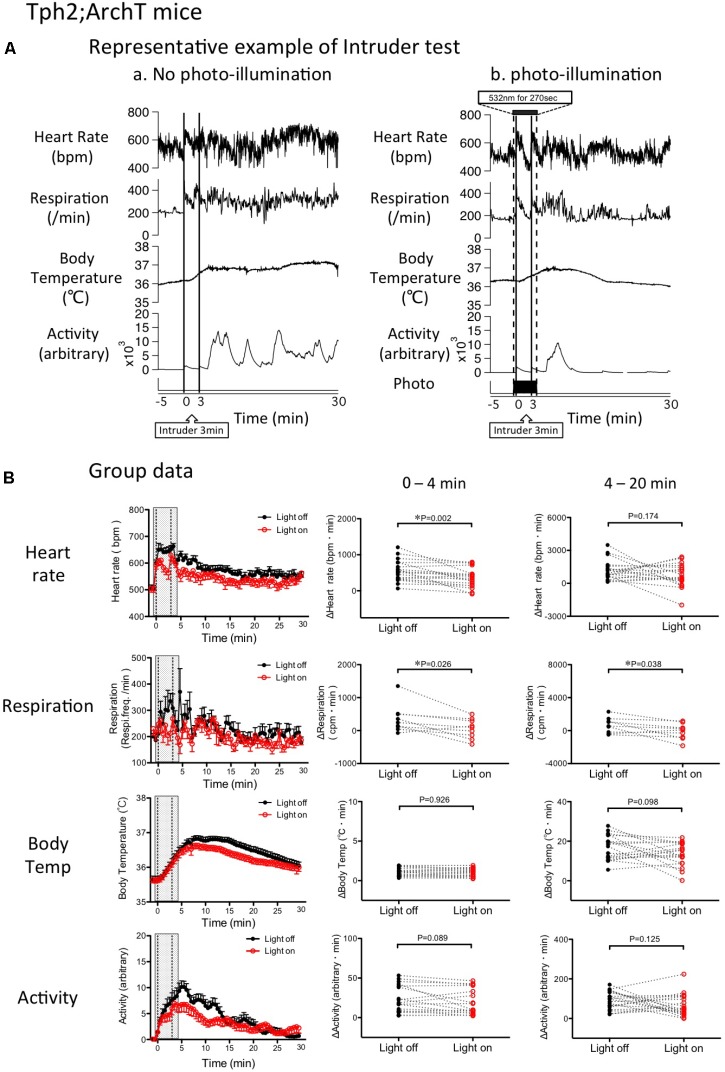
Change in intruder stress-induced physiological responses by photo inactivation of serotonin neurons in Tph2; ArchT mice. **(A)** Representative example of intruder-induced physiological responses (from top to bottom: simultaneously recorded heart rate, respiratory frequency, body temperature and locomotor activity). An intruder was introduced at time 0 and kept in the resident’s cage for 3 min. (a) Intruder test without photo-illumination. Note that the intruder stimulus caused long-lasting increases in all the measured parameters. (b) Intruder test with photo-illumination from –30 s to 240 s. **(B)** Group data of changes in physiological parameters. (Left) Time-related changes in physiological parameters. (Middle and right) Comparison between light off and on conditions. Response magnitude in heart rate, respiration rate, and body temperature during 0–4 min (Middle) or 4-20 min (Right) was calculated as area under the curve over the baseline (from –60 to 0 s). Locomotor activity was calculated as an average of the time window. Filled circles are the data obtained during the intruder test without photo-illumination. Open circles are obtained with photo-illumination. Abbreviations: bpm, beats per minute; cpm, cycles per minute. Data are shown as mean ± SEM. *N* = 19 in heart rate, body temperature, and locomotor activity. *N* = 9 in respiration.

Baseline values prior to application of the stress stimulus were not different between the “photo-illumination” group and the “without photo-illumination” group (HR = 505 ± 13 vs. 505 ± 13 bpm, Res = 196 ± 16 vs. 195 ± 15 min^-1^, BT = 35.6 ± 0.1 vs. 35.6 ± 0.0°C, Act = 0.07 ± 0.04 vs. 0.10 ± 0.04 arbitrary, paired *t*-test). During intruder stress without photo-illumination, all of the measured physiological parameters rapidly increased (**Figure 5A**). When the average value of 0–4 min was compared to the baseline value, the difference was significant for all the variables (ΔHR = 28.6 ± 4.1%, *p* < 0.001, *n* = 19; ΔRes = 47.2 ± 18.5%, *p* < 0.05, *n* = 9; ΔBT = 0.7 ± 0.1%, *p* < 0.001, *n* = 19; Act 0.10 ± 0.04 vs. 5.6 ± 1.0 (arbitrary), *p* < 0.001, *n* = 19, repeated measures ANOVA).

Possible effect of photo-inactivation of the medullary raphé serotonin neurons on changes in physiologic parameters induced by stress was assessed by calculating AUC in each parameter and comparing it using paired *t*-test. Photo inactivation of the medullary serotonergic neurons significantly attenuated tachycardia (362 ± 58 vs. 564 ± 65 bpm.min, *n* = 19, *p* = 0.002) and tachypnea (94 ± 82 vs. 361 ± 138 cpm.min, *n* = 9, *p* = 0.026) but not hyperthermia (1.0 ± 0.1 vs. 1.0 ± 0.1°C.min, *n* = 19, *p* = 0.926) or hyperlocomotion (17 ± 4 vs. 22 ± 4, arbitrary, *n* = 19, *p* = 0.089; **Figure 5B**).

During the post-illumination period (4–20 min), increases in all the physiological responses were not significantly different from those in the no photo-illumination group except for respiration (**Figure 5B**).

### Cage-Drop Test

**FIGURE 6:**
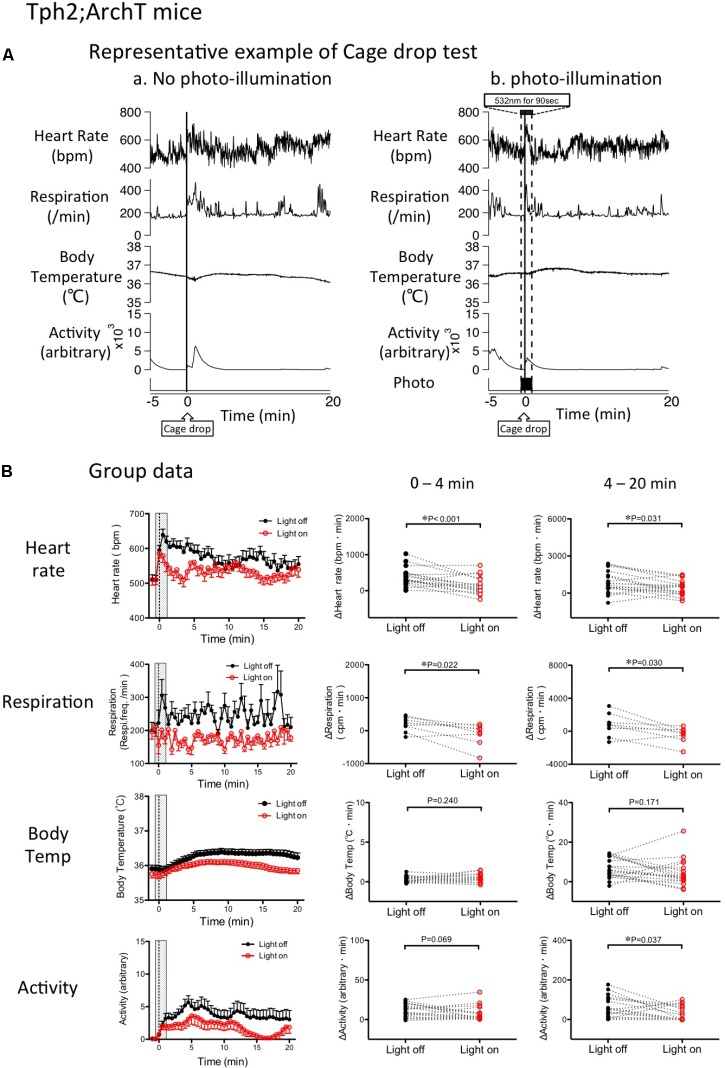
Change in cage drop-induced physiological responses by photo inactivation of serotonin neurons in Tph2; ArchT mice. **(A)** Representative example of cage-drop stress-induced physiological responses: Chart records of simultaneously recorded heart rate, respiratory frequency, body temperature, and locomotor activity. Cage-drop stimulus was applied at the 0 time point. (a) Cage-drop test without photo-illumination. Note that the cage-drop stimulus caused transient increases in all of the measured parameters. (b) Cage-drop test with photo-illumination from -30 s to 60 s. **(B)** Group data of changes in physiological parameters. (Left) Time-related changes in physiological parameters. (Middle and Right) Comparison between light off and on conditions. Response magnitude in heart rate, respiration rate, and body temperature during 0–4 min (Middle) or 4-20 min (Right) was calculated as area under the curve over the baseline (from -60 to 0 s). Locomotor activity was calculated as an average of the time window. Filled circles are the data obtained in the cage drop test without photo-illumination. Open circles are obtained with photo-illumination. Abbreviations: bpm, beats per minutes; cpm, cycles per minutes. Data are shown as mean ± SEM. *N* = 18 in heart rate, body temperature, and locomotor activity. *N* = 9 in respiration.

Baseline values prior to application of the stress stimulus were not different between the “photo-illumination” group and the “without photo-illumination” group (HR = 510 ± 14 vs. 512 ± 11 bpm, Res = 182 ± 32 vs. 203 ± 17 min^-1^, BT = 35.8 ± 0.1 vs. 35.9 ± 0.1°C, Act = 0.07 ± 0.03 vs. 0.10 ± 0.04 arbitrary, paired *t*-test). As was the case in the intruder test, all of the measured physiological parameters increased after the cage-drop stress (**Figure 6A**). The resulting values following stimulation (averaged for 240 s, same time range as in the intruder test) were significantly greater than pre-baseline levels (ΔHR = 19.5 ± 3.4%, *p* < 0.001, *n* = 18; ΔRes = 35.0 ± 12.2%, *p* < 0.05, *n* = 8; ΔBT = 0.21 ± 0.06%, *p* < 0.001, *n* = 18; Act 0.09 ± 0.04 vs. 3.0 ± 0.5 (arbitrary), *p* < 0.01, *n* = 18, repeated measures ANOVA).

Photo-inactivation of the medullary raphé serotonin neurons significantly attenuated HR and Res as compared to the no photo-illumination trial in the same animal (HR *p* = 0.001, Res *p* = 0.022, paired *t*-test; **Figure 6B**). However, the cage-drop stress-induced increases in BT (*p* = 0.240) and locomotor activity (*p* = 0.069) were not significantly affected by photo-illumination.

During the post-illumination period (4–20 min), the stress-induced increases in HR, Res, and locomotor activity were significantly smaller when compared to the no photo-illumination group (HR *p* = 0.031, Res *p* = 0.030, Act *p* = 0.037, paired *t*-test). However, BT was not affected by photo-illumination (*p* = 0.171; **Figure 6B**).

### Effect of Photo-Illumination of Medullary Raphé on Physiological Responses Induced by Stress Stimuli in Wild Type Mice

To exclude any non-specific effects from photo-illumination, such as heat, on the above-mentioned results in the Tph2-tTA; TetO-ArchT mice, we repeated the same experiment in wild type mice with an indwelling optical fiber. In this experiment, we measured HR, BT, and locomotor activity but not respiration.

Baseline values prior to application of the stress stimulus were not different between the “photo-illumination” group and the “without photo-illumination” group in both the intruder test (HR = 482 ± 21 vs. 489 ± 22 bpm, BT = 35.6 ± 0.1 vs. 35.9 ± 0.2°C, Act = 0.07 ± 0.04 vs. 0.01 ± 0.00 arbitrary, paired *t*-test) and the cage-drop test (HR = 493 ± 19 vs. 482 ± 24 bpm, BT = 35.6 ± 0.1 vs. 35.7 ± 0.1°C, Act = 0.02 ± 0.02 vs. 0.03 ± 0.01 arbitrary, paired *t*-test). These values were not statistically different (one-way ANOVA) from those in Tph2; ArchT mice (see previous sections), indicating normal basic physiology in mutant mice.

**FIGURE 7:**
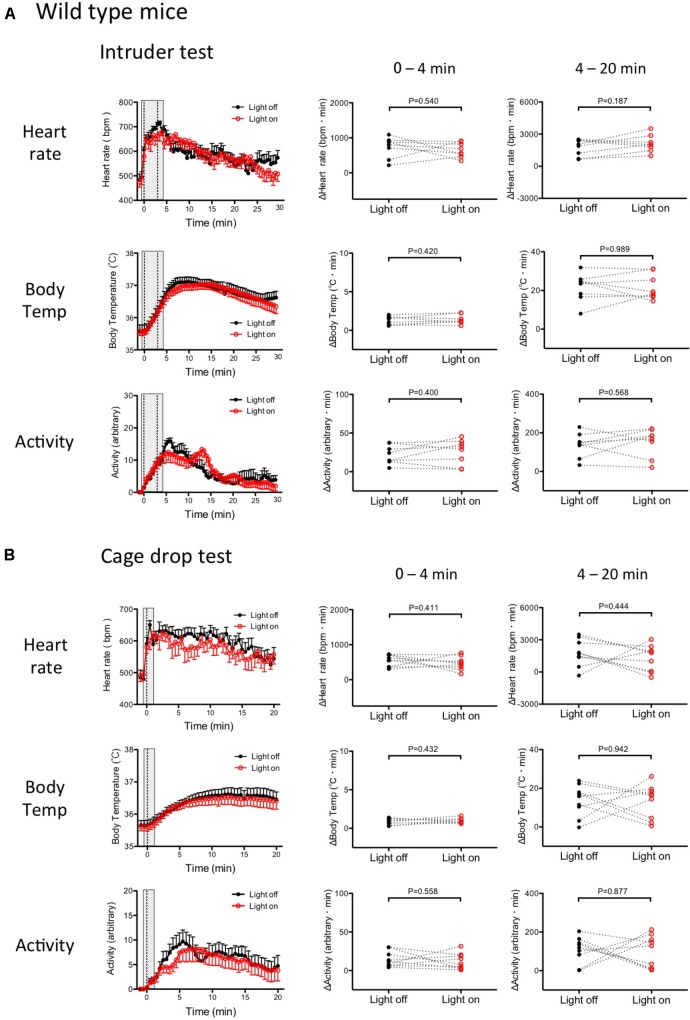
Stress-induced physiological responses in wild type mice with or without photo-illumination. **(A)** Group data of intruder-induced physiological responses. (Left) Time-related changes in physiological parameters. (Middle and Right) Comparison between light on and off conditions. Response magnitude in heart rate, respiration rate, and body temperature during 0–4 min (Middle) or 4–20 min (Right) was calculated as area under the curve over the baseline (from -60 to 0 s). Locomotor activity was calculated as an average of the time window. Filled circles are the data obtained in the drop cage test without photo-illumination. Open circles are obtained with photo-illumination. Note that there was no significant difference between the with and without photo-illumination groups. Abbreviations: bpm, beats per minutes. Data are shown as mean ± SEM. *N* = 8 in heart rate, body temperature, and locomotor activity. **(B)** Group data of the cage drop stress-induced physiological responses. (Left) Time-related changes in the physiological parameters. (Middle) Comparison between light on and off conditions (0–4 min). (Right) Comparison between light on and off conditions during post- illumination period (4–20 min). Data shown as mean ± SEM. *N* = 9 in heart rate, body temperature, and locomotor activity.

Without photo-illumination, all the measured physiological parameters rapidly increased following the intruder or cage-drop stresses. The resulting average values, during stress, were significantly greater than the pre-stress baseline levels (**Figure 7**). In the intruder test: ΔHR = 39.6 ± 6.5%, *p* < 0.001, *n* = 8; ΔBT = 0.9 ± 0.1%, *p* < 0.001, *n* = 8; Act 0.03 ± 0.02 vs. 5.5 ± 1.1 (arbitrary), *p* < 0.001, *n* = 8 (repeated measures ANOVA). In the cage-drop test: ΔHR = 29.9 ± 4.2%, *p* < 0.001, *n* = 9; ΔBT = 0.6 ± 0.1%, *p* < 0.001, *n* = 9; Act 0.03 ± 0.01 vs. 3.7 ± 0.8 (arbitrary), *p* < 0.01, *n* = 9 (repeated measures ANOVA). These values were comparable to those observed in the Tph2-tTA; TetO-ArchT mice (**Figures 5, 6**), indicating a normal stress response in the mutant mice.

As expected, both stressors (intruder and cage-drop) induced physiologic responses that were not significantly different (paired *t*-test) between the photo-illuminated trial and the not photo-illumination trial (**Figures 7A,B**).

During the post-illumination period (4–20 min), increases in all physiological responses were not significantly different from those in the no photo-illumination group (paired *t*-test) (**Figures 7A,B**). Therefore, photo-illumination by itself did not affect the defense responses in wild type mice that have no ArchT protein.

### Effect of Photo-Illumination of Medullary Raphé Serotonin Neurons on Physiological Parameters in a Resting State

**FIGURE 8:**
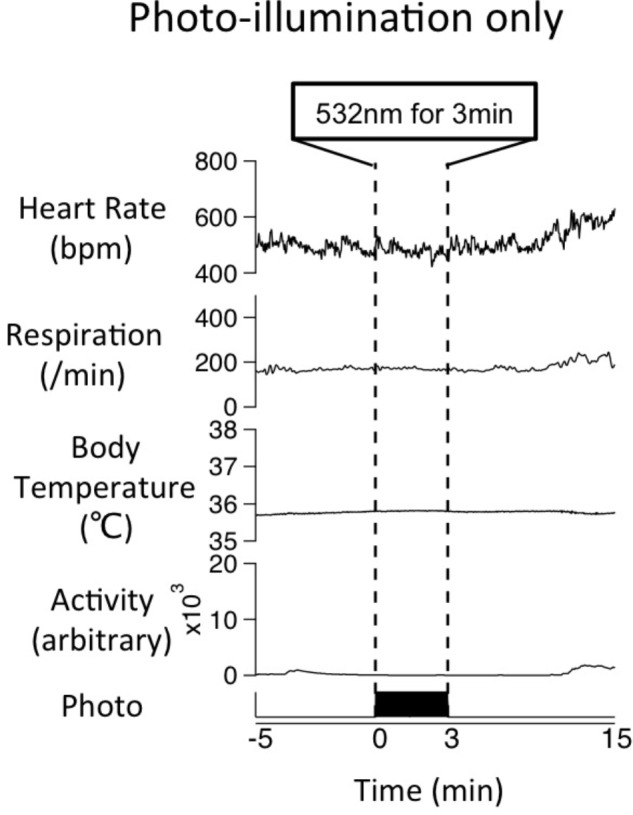
Effect of photo-illumination on baseline physiological parameters in Tph2; ArchT mouse. Averaged data of physiological parameters for 4 trials in a mouse during photo-illumination (3 min) at resting (without stress) condition. Note that little change was observed during photo-illumination. Similar results were obtained in all the four mice tested so far.

To examine the possible effect of photo-illumination on basal physiological parameters, we applied photo-illumination for 3 min when Tph2-tTA; TetO-ArchT mice were in resting condition when all physiological parameters were at a low and stable resting level. Mice were seemingly awake as their eyes were open. Photo-illumination did not affect baseline (without stress) parameters in a mutant mouse (**Figure 8**). Similar results were obtained in all the four mice tested. This experiment was performed in a subset of the mice used in the stress experiment. The mice did respond to stress and photo-illumination blunted the stress-induced responses.

## Discussion

We report for the first time that selective inactivation of serotonin neurons in the rostral medullary raphé attenuates tachycardia and tachypnea responses in the context of intruder and cage-drop stressors without affecting basal values of HR and Res. We also found that optogenetic inactivation did not affect stress-induced hyperthermia.

The optogenetic technique is unable to distinguish between cell bodies and axon terminals as both expresses transgenically driven, light sensitive proteins (Han et al., 2011). The optical illumination may activate serotonergic-nerve terminals in the medullary raphé and consequently reduce the release of serotonin. Reduced binding of serotonin to excitatory 5HT receptors in the medullary raphé may lead to inhibition of the serotonergic neurons. However, the available evidence suggests that inhibitory 5HT1A autoreceptors predominate in the medullary raphé (Wessendorf and Anderson, 1983; Pazos et al., 1985; Bayliss et al., 1997; Fay and Kubin, 2000). It is thus likely that our optical illumination caused direct inhibition of serotonin neurons in the medullary raphé.

We cannot exclude the possibility that the blunting of physiological responses to stress that we observed with photo-illumination was due, at least in part, to inhibition of non-serotonergic neurons expressing ArchT (100–93.6% = 6.4% of cells). However, our histological result showing significant decrease of pERK positive cells in the TPH positive cells by photo-illumination (**Figure 4C**) clearly support serotonergic contribution. Although the number of ArchT positive cells was not measured in this experiment, the number of non-serotonergic active cells (pERK positive and TPH negative) was not changed by photo-illumination. This observation also supports our view of serotonergic contribution.

We also cannot exclude the possibility that the blunting of physiological responses to stress that we observed with photo-illumination was due partly to inhibition of serotonergic neurons expressing ArchT in the caudal medullary raphé. Although caudal medullary raphé serotonergic neurons express less ArchT than the rostral medullary raphé serotonergic neurons (31.4 vs. 71.3%) and the position of optic fibers in the present study was 5.9–6.4 mm posterior to the bregma, corresponding to the rostral raphé, it is difficult to exclude the possible contribution of the caudal raphé serotonergic neurons. This interesting issue awaits further study.

The most pertinent finding in the study is that attenuation of stress-induced tachypnea is by and large, completely dependent on the serotonergic neurons of the rostral medullary raphé (**Figures 5, 6**). To the best of our knowledge, this is the first report showing the role of serotonergic neurons on regulation of stress-induced tachypnea. There is ample evidence to show that medullary raphé serotonin neurons are involved in respiratory activation (Veasey et al., 1995; Richerson, 2004; Corcoran et al., 2009; Ray et al., 2011). In near-complete serotonin neuron-ablated mice, the hypercapnic ventilatory response was decreased by 50%, whereas baseline ventilation and hypoxic ventilatory responses were normal (Hodges et al., 2008). Activation of serotonergic neurons in the raphé obscurus increased respiration rate; however, they were unresponsive to hypercapnia (Depuy et al., 2011). Although the possible role of serotonergic neurons in hypercapnic chemoreception is debated, their role in respiratory activation has been well established (Hodges and Richerson, 2010; Ray et al., 2011). We propose that stress is one of the physiological activators of the rostral medullary raphé serotonergic neurons involved in regulating respiration. Some of the serotonergic neurons project to the pre-Bözinger complex, which is the core of the lower brainstem respiratory network (Smith et al., 1991), and which promotes generation of respiratory motor output (Hodges and Richerson, 2008; Ptak et al., 2009). This neural projection may be involved in controlling stress-induced tachypnea.

The tachycardic response to the intruder test was not fully abolished by inhibition of the rostral medullary raphé serotonergic neurons via photo-illumination. Photo-illumination through an optic fiber may affect only the neurons located within a 1.2 mm range below the fiber tip (Han et al., 2011; Stujenske et al., 2015). Serotonergic neurons are distributed along the rostro-caudal axis in the medulla oblongata (5.5–7.5 mm posterior to the bregma, about 2 mm in the mouse) and photo-illumination may not inhibit all of these neurons. In this experiment, the center of the optic fiber was located at 5.9–6.5 mm posterior to the bregma, indicating that photo-illumination covered the majority of the rostral medullary raphé. Nevertheless, systemic injection of 5HT1A receptor agonists silences all serotonergic neurons in the medullary raphé, and also fails to induce complete disappearance of stress-induced tachycardia (Gothert, 1990; Ngampramuan et al., 2008). We thus hypothesize that the persistent tachycardia is mediated by non-serotoninergic neurons.

In the present study, stress-induced hyperactivity and hyperthermia were not attenuated by inactivation of serotonergic neurons in the medullary raphé – except for hyperactivity during the later phase in the cage-drop test. As general inactivation of neurons in the medullary raphé (with muscimol, a GABA-A agonist) inhibits a stress-induced hyperthermia and hyperactivity (Zaretsky et al., 2003a; Kataoka et al., 2014), we can infer that stress-induced hyperthermia and hyperactivity is mediated by systems other than serotonin neurons in the medullary raphé. Before this conclusion can be made, however, a few points need to be addressed. As discussed previously, it is possible that not all of the serotonergic neurons in the medullary raphé were covered under our photo-illumination. Also, a short photo-illumination period may not be sufficient to attenuate stress-induced hyperthermia. More prolonged inactivation of serotonergic neurons with chemogenetics may be useful (Armbruster et al., 2007; Alexander et al., 2009; Inutsuka et al., 2014). Further evidence by Ray et al. (2011) reported that DREADDs induced inactivation of a subset of medullary raphé serotonin neurons produced a decrease in basal BT. This report conflicts with the current results and highlights the possible existence of various serotonin neuron subpopulations within the medullary raphé.

The stress-induced hyperthermic response is largely due to heat production in brown adipose tissue (BAT; Lkhagvasuren et al., 2011; Mohammed et al., 2014). Serotonergic neurons in the medullary raphé send axons to the intermediolateral nucleus (IML) and control outflow to the BAT (Cao et al., 2004; Nakamura et al., 2004). Serotonergic receptors are expressed within the IML (Marlier et al., 1991; Cornea-Hebert et al., 1999). Microinjection of serotonin into the IML elicits BAT thermogenesis (Madden and Morrison, 2006). These reports suggest the possible involvement of bulbospinal serotonin neurons in stress-induced hyperthermia. Interestingly, the BAT thermogenesis with serotonin in the IML has a long onset latency of over 20 min, whilst with glutamate receptor agonists, it has a short latency (Madden and Morrison, 2006). Data analyzed during the photo-illumination period (0–4 min) as well as the later phase (4–20 min after the onset of stress) found that there was no difference in BT – even in the later phase. Therefore, contribution of the bulbospinal serotonin neurons in the medullary raphé to stress-induced hyperthermia, seems minimal, if any. Although we cannot determine possible neurotransmitter for stress-induced hyperthermia other than serotonin from the current study, glutamatergic neurotransmission is a plausible alternative. Blockade of glutamate receptors in the IML eliminates BAT thermogenesis evoked by stimulation of the rostral medullary raphé (Nakamura et al., 2004). This mechanism can be applied to stress-induced thermal responses, because glutamatergic (VGLUT3-expressing) sympathetic premotor neurons, as well as serotonergic neurons in the rostral medullary raphe has been shown to be activated by psychological stress (Lkhagvasuren et al., 2011).

Multiple studies have shown that inhibition of medullary raphé neurons does not affect resting HR and blood pressure (Mccall and Harris, 1987; Samuels et al., 2002; Cao et al., 2004). While evidence suggests that the medullary raphé does not play a significant cardiovascular role in the context of a resting state, it has been suggested that it mediates an active cardiovascular response on a background of stress. The present study supports this view. We have found that the subpopulation of serotonergic neurons in the medullary raphé, which have little involvement in the basal determination of Res and HR, mediate tachycardia and tachypnea during stressful conditions.

## Author Contributions

YI, YO, and TK designed the study and wrote the manuscript. All authors conducted the study, analyzed the data, and approved the final version of the manuscript.

## Conflict of Interest Statement

The authors declare that the research was conducted in the absence of any commercial or financial relationships that could be construed as a potential conflict of interest. The reviewer KN declared a shared affiliation, with no collaboration, with one of the authors AY to the handling Editor.
